# Bias-corrected estimator for intraclass correlation coefficient in the balanced one-way random effects model

**DOI:** 10.1186/1471-2288-12-126

**Published:** 2012-08-20

**Authors:** Eshetu G Atenafu, Jemila S Hamid, Teresa To, Andrew R Willan, Brian M Feldman, Joseph Beyene

**Affiliations:** 1 Princess Margaret Hospital, Toronto, Canada; 2 Department of Clinical Epidemiology and Biostatistics, McMaster University, Hamilton, Canada; 3 Child Health Evaluative Sciences Hospital for Sick Children, Toronto, Canada; 4 Division of Biostatistics, Dalla Lana School of Public Health, University of Toronto, Toronto, Canada; 5 Departments of Pediatrics, Health Policy and Management, University of Toronto, Toronto, Canada

## Abstract

**Background:**

Intraclass correlation coefficients (ICCs) are used in a wide range of applications. However, most commonly used estimators for the ICC are known to be subject to bias.

**Methods:**

Using second order Taylor series expansion, we propose a new bias-corrected estimator for one type of intraclass correlation coefficient, for the ICC that arises in the context of the balanced one-way random effects model. A simulation study is performed to assess the performance of the proposed estimator. Data have been generated under normal as well as non-normal scenarios.

**Results:**

Our simulation results show that the new estimator has reduced bias compared to the least square estimator which is often referred to as the conventional or analytical estimator. The results also show marked bias reduction both in normal and non-normal data scenarios. In particular, our estimator outperforms the analytical estimator in a non-normal setting producing estimates that are very close to the true ICC values.

**Conclusions:**

The proposed bias-corrected estimator for the ICC from a one-way random effects analysis of variance model appears to perform well in the scenarios we considered in this paper and can be used as a motivation to construct bias-corrected estimators for other types of ICCs that arise in more complex scenarios. It would also be interesting to investigate the bias-variance trade-off.

## Background

The intraclass correlation coefficient (ICC), often denoted by *ρ*, was first introduced by Fisher [1] to study the familial resemblance between siblings. Since then it has obtained a wide range of applications in many areas such as psychology, epidemiology, genetics and genomics. See Donner [2] for an extensive review of inference procedures. In psychology, it plays a fundamental role in studying inter-rater reliability [3,4]. It is used as a measure of heritability in classical genetic linkage studies to quantify the proportion of variance in traits of interest explained by genetic factors [5]. Intraclass correlation obtained from genome-wide association data has recently been used to provide a better estimate of heritability [6] . Sensitivity analysis is another application where *ρ* may be used as a means of investigating the effectiveness of an experimental treatment [7]. The intraclass correlation has also found some interesting application in genomics where it has been used to assess methodological and biological variations in DNA microarray analysis [8].

The intraclass correlation coefficient also plays a key role in study design such as design of cluster randomized trials where it is traditionally used to quantify the degree of similarity between individuals within clusters [9,10].

Over the last decade, ICCs have received more attention in the literature and there has been an increasing awareness and appreciation of methodological issues related to these indices [11-13].

The most fundamental interpretation of ICCs is as a measure of the proportion of variance of a given outcome variable explained by a factor of interest in an analysis of variance model where it measures the relative homogeneity within groups [14,15]. The first and essential step, therefore, is to specify an appropriate analysis of variance (ANOVA) model that best describes the study. The choice of the model is dictated by the specific situation defined by the experimental design and conceptual intent of the study [15]. Moreover, various forms of ICCs arise depending on the chosen model and the nature of the study [16,17].

For reasons mentioned above, inference procedures for *ρ* are closely related to the more general statistical problem of variance components [14,18]. It is well known that estimation and hypothesis testing procedures for ICCs are, in general, sensitive to the assumption of normality and are subject to unstable variance [1,19]. One, therefore, needs to consider normalizing and variance-stabilizing transformations on the basis of the rate of convergence to normality when constructing confidence intervals for the ICC. One of the well known and most commonly used normalization technique is Fisher’s Z transformation [1]. Other types of transformations have also been considered for the intraclass correlation coefficient [19,20].

Another important issue concerning ICCs is bias [21,22]. The two most commonly used estimators, maximum likelihood and least square estimators, are known to be negatively biased. Although a Minimum Variance Unbiased (MVU) estimator for the intraclass correlation coefficient under two normal distributions is derived by [23], use of this estimator has been hindered because of absence of a closed form. Consequently, the MVU estimator is less widely recognized while the least square and maximum likelihood estimators are well-known. A computationally intensive FORTRAN subroutine is provided by Donoghue and Collins (1990).

The purpose of this paper is, therefore, to provide a bias-corrected estimator for the intraclass correlation coefficient which is much simpler to compute and hence useful in practice. We consider a particular type of ICC in which we consider the estimation problem for ICC resulting from a one-way random effects analysis of variance model. We approximate the bias using a second-order Taylor series expansion and adjust the estimator to reduce the bias.

The paper is organized as follows. We provide a brief background about the one-way random effects model and define the particular ICC of interest in Section “Methods”. In Section “Bias-corrected estimator for the intraclass correlation coefficient”, we propose a technique for approximating the bias resulting from the conventional estimator of *ρ* and we derive a new bias-corrected estimator for the parameter. We present simulation results in Section “Simulation Study” and provide a brief discussion in Section “Discussion”. Finally an Appendix consisting of some technical results is given at the end of the paper.

## Methods

Consider *n* targets measured by k raters (instruments, judges etc.). A commonly used model for inferences concerning the intraclass correlation coefficient is the one-way random effects analysis of variance model where the *j*^*th*^ target measurement by the *i*^*th *^rater (j = 1, 2, …, *n* ; i = 1, 2, …, *k*) can be described as

(1) $$Y_{\text{ij}}=\mu +a_{i}+e_{\text{ij}}$$

where it is assumed that $a_{i}∼N(0,\sigma_{T}^{2}),\;\;\;e_{\text{ij}}∼N(0,\sigma_{e}^{2})$.

The total sum of squares (Total SS) for the above model can be decomposed into two independent components as follows

(2) $$\begin{array}{l} \overset{n}{\underset{i=1}{\sum }}\overset{k}{\underset{j=1}{\sum }}{\left(Y_{\text{ij}}-\bar{Y}.\right)}^{2}=\overset{n}{\underset{i=1}{\sum }}\overset{k}{\underset{j=1}{\sum }}{\left(Y_{\text{ij}}-{\bar{Y}}_{\mathrm{i.}}\right)}^{2}+\overset{n}{\underset{i=1}{\sum }}\overset{k}{\underset{j=1}{\sum }}{\left({\bar{Y}}_{\mathrm{i.}}-\bar{Y}.\right)}^{2}\\ \;\;\text{Total}\;\text{SS}=\text{SSE}+\text{SSB}, \end{array}$$

where SSE and SSB represent the within and between target sum of squares, respectively. The above decomposition is summarized in the analysis of variance table provided in Table 1. The table shows the source of variation, degrees of freedom (df), sums of squares (SS), mean square (MS), and expected mean square. We refer the reader to any standard analysis of variance text book [24] for details about this model.

**Table 1:** Analysis of variance table for one-way random effects model

**Source of Variation**	**df**	**SS**	**MS=SS/df**	**Expected MS**
Between Targets	n-1	SSB	BMS	$k\sigma_{T}^{2}+\sigma_{e}^{2}$
Within Targets	n(k-1)	SSE	EMS	$\sigma_{e}^{2}$

The intraclass correlation coefficient for the model in (1) is defined as

(3) $$\rho =\frac{\sigma_{T}^{2}}{\sigma_{T}^{2}+\sigma_{e}^{2}}.$$

The most commonly used estimator for *ρ*, which is sometimes referred to as the analytical estimator, is given by

(4) $$\hat{\rho }=\frac{{\hat{\sigma }}_{T}^{2}}{{\hat{\sigma }}_{T}^{2}+{\hat{\sigma }}_{e}^{2}}=\frac{\text{BMS}-\text{EMS}}{\text{BMS}+(k-1)*\text{EMS}}.$$

Note that, 

• EMS is an unbiased estimator of $\sigma_{e}^{2}$

• (BMS-EMS)/k is an unbiased estimator of $\sigma_{T}^{2}$

Although the estimator in (4) is a ratio of unbiased estimators, it need not necessarily be unbiased itself. We consider the bias resulting from this estimator in the next section and provide a new bias-corrected estimator for the intraclass correlation coefficient.

### Bias-corrected estimator for the intraclass correlation coefficient

Consider the intraclass correlation coefficient defined in (3) and re-write it as

(5) $$\rho =\frac{\frac{\sigma_{T}^{2}}{\sigma_{e}^{2}}}{1+\frac{\sigma_{T}^{2}}{\sigma_{e}^{2}}}=\frac{F}{1+F}.$$

An unbiased estimator for F is provided in the following theorem, which is useful in approximating the bias for estimating intraclass correlation coefficient. The variance of is also given in the theorem. The theorem has been considered by [25] in a different context.

#### Theorem 1

Consider $F=\frac{\sigma_{T}^{2}}{\sigma_{e}^{2}}$ in equation (5), then

$$\hat{F}=\frac{\left[n(k-1)-2]*\text{SSB}/\text{SSE}-(n-1\right)}{k(n-1)}$$

is an unbiased estimator for F. Moreover, its variance is given by

$$\begin{array}{ll} \text{Var}(\hat{F})=\left[\frac{n(k-1)-2}{k^{2}(n-1)}\right]\\ \;\;\times \left[\frac{n+1}{n(k-1)-4}-\frac{n-1}{n(k-1)-2}\right]{\left(\text{kF}+1\right)}^{2}\; & \; \end{array}$$

A proof of the theorem is provided in the Appendix.

Let us now consider the following estimator for the intraclass correlation coefficient which is derived by substituting the unbiased estimator for F given in Theorem 1.

(6) $$\tilde{\rho }=\frac{\hat{F}}{\hat{F}+1},$$

As mentioned earlier, the estimator, $\tilde{\rho }$, in (6) need not be unbiased although it is a function of unbiased estimators. In fact, the bias is always negative and depends on the degree of correlation and the design size and balance [21].

Now consider (5) and apply log transformation on both sides. The equation reduces to

logρ=logF−log(F+1).

An estimator for log*ρ*, which is obtained by substituting F by its unbiased estimator, can be given as

$$\hat{\text{log}\rho }=\text{log}\hat{F}-\text{log}(\hat{F}+1).$$

Note that the above estimator is equivalent to log $\tilde{\rho }$. The bias, in log scale can, therefore, be given as

$$\begin{array}{ll} E[\text{log}\tilde{\rho }-\text{log}\rho ]=E[\text{log}\hat{F}-\text{log}F]\\ \;\;\;-E[\text{log}(\hat{F}+1)-\text{log}(F+1)].\; & \; \end{array}$$

Using a second order Taylor series approximation, we have

$$\begin{array}{ll} \;\;\text{log}\hat{F}\approx \text{log}F+\frac{1}{F}(\hat{F}-F)-\frac{1}{2F^{2}}{\left(\hat{F}-F\right)}^{2}\\ \text{log}(\hat{F}+1)\approx \text{log}(F+1)+\frac{1}{F+1}(\hat{F}-F)\\ \;\;-\frac{1}{2{\left(F+1\right)}^{2}}{\left(\hat{F}-F\right)}^{2}.\; & \; \end{array}$$

Consequently, the bias can be approximated as

(7) $$E[\text{log}\tilde{\rho }-\text{log}\rho ]\approx \frac{-1}{2}\left[\frac{1}{F^{2}}-\frac{1}{{\left(F+1\right)}^{2}}\right]\text{Var}(\hat{F})$$

It is important to note that the above approximation for the bias is always negative indicating that we are correcting the estimator from the right direction. A bias-corrected estimator for log *ρ* is obtained by adjusting for the bias given in (7) and is given by

$${\hat{\text{log}\rho }}_{\text{bc}}=\text{log}\tilde{\rho }+\frac{1}{2}\left[\frac{1}{{\hat{F}}^{2}}-\frac{1}{{\left(\hat{F}+1\right)}^{2}}\right]\hat{\text{Var}}(\hat{F}),$$

where the subscript bc indicates that the estimator is bias corrected. $\hat{\text{Var}}(\hat{F})$ is an estimator for the $\text{Var}(\hat{F})$ obtained by substituting F in $\text{Var}(\hat{F})$ by $\hat{F}$. We obtain a bias-corrected estimator for the intraclass correlation coefficient by transforming the above equation back to the original scale, and it is given by

(8) $${\hat{\rho }}_{\text{bc}}=\tilde{\rho }*\exp\left\{\frac{1}{2}\left[\frac{1}{{\hat{F}}^{2}}-\frac{1}{{\left(\hat{F}+1\right)}^{2}}\right]\hat{\text{Var}}\hat{F}\right\}$$

In situations where F is small, we consider Taylor expansion of 1−*ρ* instead. Consequently,

$${\hat{\rho }}_{\text{bc}}=1-(1-\tilde{\rho })*\exp\left\{-\frac{1}{2{\left(\hat{F}+1\right)}^{2}}\hat{\text{Var}}(\hat{F})\right\}.$$

It can be shown using our approximation that the bias in general decreases as the degree of correlation moves away from 0.5. That is, the bias is small for both weak and strong correlations. Our simulation results confirm that the bias resulting from the analytical estimator is indeed small when the true value of *ρ*is small (weak correlation) or large (strong correlation) (see Section “Discussion” for more details). As a result, we expect the performance of the conventional estimator to improve in such cases. In fact, previous simulation results have also showed that the analytical estimator, $\hat{\rho }$, performs well for small values of *ρ *both for normal and non-normal data (e.g., producing confidence intervals close to the nominal level [10]. Our simulation results show that the estimators we proposed in this paper provide a considerable bias reduction even under such circumstances (see Section “Discussion” for details).

### Simulation Study

We carried out extensive simulations to evaluate the performance of our bias-corrected estimator $\left({\tilde{\rho }}_{\text{bc}}\right)$. The bias resulting from our estimator is compared with bias from the conventional (analytical) estimator using normal as well as non-normal data. It is to be recalled that we based the Taylor expansion around $\tilde{\rho }$ which a variant of the conventional estimator. We have, therefore, provided the bias resulting from this estimator for comparison purposes.

### Simulation Design

Data were simulated as in [10], with slight modifications to the number of configurations that were allowed to vary. We used 3x8x2 design instead of their 3x4x2 configuration. That is, we considered 3 cluster sizes (10,30,50); 8 intraclass correlation coefficients (0.2, 0.3, 0.4, 0.5, 0.6, 0.7, 0.8, 0.9) instead of only 4 true ICC values used in [10]; and two types of data distributions (normal versus non-normal), for a total of 48 simulation configurations.

For normal outcome, data were simulated according to the framework of the one-way random effects model described in Section “Methods”. They were generated as the sum of two independent random variables $a_{i}∼N(0,\sigma_{T}^{2})$ and $e_{\text{ij}}∼N(0,\sigma_{e}^{2})$. When simulating non-normal outcome, the *e*_*ij*_ alone were generated from a normal distribution with the *a*_*i *_generated from a Gamma distribution with shape parameter *α *= 1.67 and scale parameter $\beta =\frac{\sqrt{\text{Var}}}{\alpha }$ so that skewness of the distribution will be $\frac{2}{\alpha }=1.2$ and a kurtosis coefficient of $\frac{6}{\alpha }=4$. The skewness and kurtosis coefficients represent marked deviation from normality. The skewness and kurtosis coefficients for a normally distributed random variable are 0 and 3, respectively.

Without loss of generality, the overall mean was fixed at 10. Moreover, the sum of the variance components was constrained to be 1000, for both normal and non-normal data sets. The two variances, $\sigma_{T}^{2}$ and $\sigma_{e}^{2}$, were then systematically manipulated to generate data from a population with the required ICC value using the following relationship

$$\begin{array}{lll} & \sigma_{T}^{2}+\sigma_{e}^{2}=1000,\;\;\rho =\frac{\sigma_{T}^{2}}{\sigma_{T}^{2}+\sigma_{e}^{2}}\;\;\;\Rightarrow \;\;\;\sigma_{T}^{2}=1000*\rho \;\;\;\;\; & \;\\ & \text{and}\;\;\;\;\sigma_{T}^{2}=1000-\sigma_{e}^{2}\; & \; \end{array}$$

We simulated 5000 replications of data for each of the 48 scenarios. Different seeds were used for the random number generator at each replication while keeping it the same across different methods.

## Results

**Figure 1:**
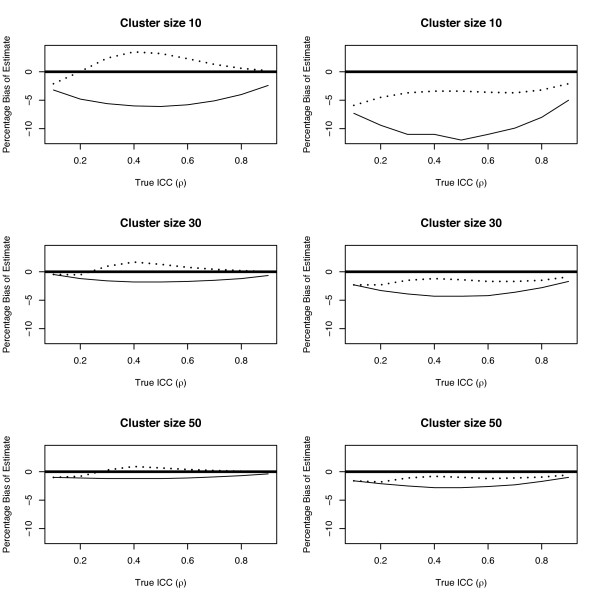
Plot of the percentage bias against the true value of ICC for the normal (left panel) and non-normal (right panel) data sets, where solid line and dashed line represent the analytical and bias-corrected estimators, respectively.

Estimates of ICC along with percentage of bias for non-normal and normal data, averaged over 5000 simulations, are summarized in Table 2 and Table 3, respectively. As expected, the bias resulting from the analytical estimator is negative both for normal and non-normal data sets. Moreover, the bias gets smaller as the true value of *ρ* moves further away from 0.5 (see Figure 1). Furthermore, the biases resulting from the conventional estimator ( $\hat{\rho }$) and its variant ( $\tilde{\rho }$) for the non-normal sample are larger relative to the normal sample indicating that the estimator is sensitive to the normality assumption. The difference is much larger for small cluster sizes. For cluster size 10 from the non-normal data with moderate correlation (*ρ *= 0.4,0.5,0.6), for example, the analytical estimator and its variant gave about 12% and 13% bias, respectively. However, the corresponding biases for normal data with the same cluster size and similar *ρ* values are 6% and 7%, respectively. It is, therefore, not recommended to use the analytical estimator for non-normal data, especially when there is moderate correlation. It is also important to note that the bias resulting from $\hat{\rho }$ is uniformly smaller than that of $\tilde{\rho }$ where the difference is much larger for small cluster sizes.

**Table 2:** Simulation results for the non-normal data

		**Estimate**			**% Bias**		
**Clusters**	**ICC**	$\hat{\rho }$	$\tilde{\rho }$	${\tilde{\rho }}_{\mathrm{bc}}$	$\hat{\rho }$	$\tilde{\rho }$	${\tilde{\rho }}_{\mathrm{bc}}$
10	0.1	0.0927	0.0891	0.0941	-7.3	-11.0	-5.9
	0.2	0.1813	0.1768	0.1911	-9.4	-12.0	-4.5
	0.3	0.2678	0.2627	0.2890	-11.0	-12.0	-3.7
	0.4	0.3543	0.3488	0.3865	-11.0	-13.0	-3.4
	0.5	0.4423	0.4367	0.4830	-12.0	-13.0	-3.4
	0.6	0.5337	0.5282	0.5785	-11.0	-12.0	-3.6
	0.7	0.6306	0.6256	0.6744	-9.9	-11.0	-3.7
	0.8	0.7360	0.7319	0.7743	-8.0	-8.5	-3.2
	0.9	0.8552	0.8526	0.8809	-5.0	-5.3	-2.1
30	0.1	0.0977	0.0964	0.0977	-2.3	-3.6	-2.3
	0.2	0.1935	0.1919	0.1954	-3.3	-4.1	-2.3
	0.3	0.2883	0.2865	0.2954	-3.9	-4.5	-1.5
	0.4	0.3830	0.3810	0.3951	-4.3	-4.8	-1.2
	0.5	0.4783	0.4763	0.4929	-4.3	-4.7	-1.4
	0.6	0.5751	0.5733	0.5899	-4.2	-4.4	-1.7
	0.7	0.6745	0.6728	0.6880	-3.6	-3.9	-1.7
	0.8	0.7773	0.7760	0.7883	-2.8	-3 .0	-1.5
	0.9	0.8851	0.8844	0.8919	-1.7	-1.7	-0.9
50	0.1	0.0984	0.0977	0.0984	-1.6	-2.3	-1.6
	0.2	0.1957	0.1947	0.1965	-2.1	-2.6	-1.8
	0.3	0.2924	0.2912	0.2966	-2.5	-2.9	-1.1
	0.4	0.3890	0.3878	0.3968	-2.8	-3.1	-0.8
	0.5	0.4862	0.4850	0.4951	-2.8	-3.0	-1.0
	0.6	0.5844	0.5832	0.5931	-2.6	-2.8	-1.2
	0.7	0.6842	0.6832	0.6921	-2.3	-2.4	-1.1
	0.8	0.7861	0.7854	0.7925	-1.7	-1.8	-0.9
	0.9	0.8911	0.8907	0.8949	-1.0	-1.0	-0.6

**Table 3:** Simulation results for the normal data

		**Estimate**			**% Bias**		
**Clusters**	**ICC**	$\hat{\rho }$	$\tilde{\rho }$	${\tilde{\rho }}_{\mathrm{bc}}$	$\hat{\rho }$	$\tilde{\rho }$	${\tilde{\rho }}_{\mathrm{bc}}$
10	0.1	0.0969	0.0932	0.0979	-3.2	-6.8	-2.1
	0.2	0.1905	0.1858	0.2001	-4.8	-7.1	0.1
	0.3	0.2832	0.2778	0.3071	-5.6	-7.4	2.4
	0.4	0.3759	0.3701	0.4140	-6.0	-7.5	3.5
	0.5	0.4696	0.4637	0.5158	-6.1	-7.3	3.2
	0.6	0.5652	0.5596	0.6135	-5.8	-6.7	2.3
	0.7	0.6641	0.6591	0.7094	-5.1	-5.8	1.3
	0.8	0.7677	0.7638	0.8049	-4.0	-4.5	0.6
	0.9	0.8784	0.8760	0.9016	-2.4	-2.7	0.2
30	0.1	0.0995	0.0983	0.0995	-0.5	-1.7	-0.5
	0.2	0.1976	0.1960	0.1989	-1.2	-2.0	-0.6
	0.3	0.2953	0.2934	0.3029	-1.6	-2.2	1.0
	0.4	0.3929	0.3909	0.4067	-1.8	-2.3	1.7
	0.5	0.4910	0.4889	0.5063	-1.8	-2.2	1.3
	0.6	0.5897	0.5878	0.6047	-1.7	-2.0	0.8
	0.7	0.6895	0.6878	0.7030	-1.5	-1.7	0.4
	0.8	0.7908	0.7895	0.8015	-1.2	-1.3	0.2
	0.9	0.8941	0.8934	0.9005	-0.7	-0.7	0.1
50	0.1	0.0990	0.0983	0.0990	-1.0	-1.7	-1.0
	0.2	0.1978	0.1969	0.1984	-1.1	-1.6	-0.8
	0.3	0.2965	0.2954	0.3009	-1.2	-1.5	0.3
	0.4	0.3952	0.3940	0.4037	-1.2	-1.5	0.9
	0.5	0.4942	0.4929	0.5033	-1.2	-1.4	0.7
	0.6	0.5935	0.5924	0.6024	-1.1	-1.3	0.4
	0.7	0.6936	0.6926	0.7015	-0.9	-1.1	0.2
	0.8	0.7945	0.7938	0.8008	-0.7	-0.8	0.1
	0.9	0.8966	0.8961	0.9002	-0.4	-0.4	0.0

In general, a considerable bias reduction has been obtained by using our bias-corrected estimator. This is true for all values of *ρ*and all cluster sizes although the improvement is much larger for moderate correlations (see Figure 1). Moreover, the improvement obtained for the non-normal sample is relatively larger than that obtained for the normal sample. For the non-normal sample, for instance, using the conventional estimator resulted in 12% bias whereas only 3.4% bias was obtained from our estimator for cluster size 10 and moderate correlations. For the normal sample and the same scenario, biases resulting from the conventional estimator and the bias-corrected estimator are 6% and 3%, respectively. Improvements are also obtained for small or large *ρ* values, that is, in situations where the bias from the conventional estimator is small. For instance, for cluster size 10 from the non-normal sample with *ρ *= 0.2, 9.4% and 12% biases were obtained using $\hat{\rho }$ and $\tilde{\rho }$, respectively, whereas only 4.5% bias was observed for our estimator. For the normal sample with the same cluster size and correlation, $\hat{\rho }$ and $\tilde{\rho }$ resulted in 4.8% and 7.1% biases. In this situation, the bias reduced to 0.05% when using our bias corrected estimator. Similar statements can be made for large ICC values.

## Discussion

The intraclass correlation coefficient (ICC) has widespread applications from measuring heritability in genetic studies to measuring reliability, consistency and agreement of measurements in a host of clinical, biomedical and psychosocial areas. The ICC has important role in study design and sample size calculations as well. For instance, designs of family-based genetic studies can be greatly impacted by the estimated ICC (often referred to as the coefficient of heritability in the genetics literature). Similarly, trials involving clustering of some degree (e.g., longitudinal study design, multilevel models, cluster randomized trials, etc.) will be influenced to various extent by the magnitude of the intraclass correlation coefficient. Because ICC estimates have great implications to design considerations, statistical analysis as well as interpretation of study findings, it is critical to use an estimator with minimal bias.

In this paper, we proposed a new bias-corrected estimator for one type of intraclass correlation coefficient. We used a variant of the conventional estimator (ANOVA estimator) and applied Taylor series expansion to approximate the bias. The approximate bias was then used and a new adjusted estimator is proposed. The bias-corrected estimator proposed in this paper is much simpler to compute than the minimum variance unbiased (MVU) estimator of Olkin and Pratt [23]. Moreover, our simulation study shows that our estimator outperforms the conventional estimator by providing a substantial decrease in the bias. For small cluster sizes from normal data, however, a positive bias was introduced although the percentage bias resulted from our estimator is still smaller than that of the ANOVA estimator. This might be improved by using second order Taylor series expansion instead of using only the first order adopted in this paper.

## Conclusion

We considered a particular type of intraclass correlation coefficient that arises from a one-way random effects analysis of variance model, although the method can be extended to provide bias-corrected estimators for other types of ICCs. Furthermore, the current paper is focused on bias reduction in a balanced data setting, and we plan to investigate other optimality measures as well as the performance of the bias-corrected method for unbalanced data when the number of observations differ from cluster to cluster. Finally, we would like to highlight that ICCs are subject to different interpretations, so the user should apply the various ICCs with caution [17,26-28].

## Appendix

Recall from the one-way ANOVA model $\frac{\text{SSB}}{k\sigma_{T}^{2}+\sigma_{e}^{2}}∼\chi^{2}(n-1)$. Therefore, one can easily show that $E[\text{SSB}]=(n-1)(k\sigma_{T}^{2}+\sigma_{e}^{2})$*and*$E{\left[\text{SSB}\right]}^{2}=(n-1)(n+1){\left[k\sigma_{T}^{2}+\sigma_{e}^{2}\right]}^{2}$ Moreover, $\text{SSE}/\sigma_{e}^{2}\;\;∼\;\;\chi^{2}(n(k-1))=\text{Gamma}\left(\frac{n(k-1)}{2},2\right)$. Consequently,

$$\begin{array}{lll} E\left[\frac{1}{\frac{\text{SSE}}{\sigma_{e}^{2}}}\right] & =E\left[\frac{\sigma_{e}^{2}}{\text{SSE}}\right]\frac{2^{-1}\Gamma (n(k-1)/2-1)}{\Gamma (n(k-1)/2)}\; & \;\\ & =\frac{1}{n(k-1)-2}\; & \; \end{array}$$

Which implies that $E\left[\frac{1}{\text{SSE}}\right]=\frac{1}{\sigma_{e}^{2}\left[n(k-1)-2\right]}$.

Moreover,

$$\begin{array}{lll} & E{\left[\frac{\text{SSE}}{\sigma_{e}^{2}}\right]}^{-2}\frac{2^{-2}\Gamma (n(k-1)/2-2)}{\Gamma (n(k-1)/2)}\; & \;\\ = & \frac{1}{\left[n(k-1)-2][n(k-1)-4\right]}\; & \; \end{array}$$

As a result we have,

$$E{\left[\frac{1}{\text{SSE}}\right]}^{2}=\frac{1}{{\left(\sigma_{e}^{2}\right)}^{2}[n(k-1)-2][n(k-1)-4]}$$

Now applying results from [24], we get

$$\begin{array}{lll} E\left[\frac{\text{SSB}}{\text{SSE}}\right] & =E\left[\text{SSB}\left(\frac{1}{\text{SSE}}\right)\right]=E[\text{SSB}]*E\left[\frac{1}{\text{SSE}}\right]\; & \;\\ & =\frac{\left(n-1)(k\sigma_{T}^{2}+\sigma_{e}^{2}\right)}{[n(k-1)-2]\sigma_{e}^{2}}\; & \;\\ & =\frac{\left(n-1\right)}{n(k-1)-2}\left[k\frac{\sigma_{T}^{2}}{\sigma_{e}^{2}}+1\right]\; & \; \end{array}$$

After a simple algebraic manipulations, we can show that

$$E[\hat{F}]=\frac{1}{k}\left[\frac{n(k-1)-2}{n-1}E\left(\frac{\text{SSB}}{\text{SSE}}\right)-1\right]=\frac{\sigma_{T}^{2}}{\sigma_{e}^{2}}$$

which proves the first part of the theorem.

Now consider,

$$\begin{array}{lll} E{\left[\frac{\text{SSB}}{\text{SSE}}\right]}^{2} & =E{\left[\text{SSB}\right]}^{2}*E{\left[\frac{1}{\text{SSE}}\right]}^{2}\; & \;\\ & =\frac{\left(n-1)(n+1\right)}{\left[n(k-1)-2][n(k-1)-4\right]}{\left(\frac{k\sigma_{T}^{2}+\sigma_{e}^{2}}{\sigma_{e}^{2}}\right)}^{2}\; & \; \end{array}$$

Consequently,

$$\begin{array}{lll} \text{Var}\left[\frac{\text{SSB}}{\text{SSE}}\right] & =\left[\frac{\left(n-1)(n+1\right)}{\left[n(k-1)-2][n(k-1)-4\right]}\right)\; & \;\\ & \;\left(-{\left(\frac{n-1}{n(k-1)-2}\right)}^{2}\right]{\left(\frac{k\sigma_{T}^{2}+\sigma_{e}^{2}}{\sigma_{e}^{2}}\right)}^{2}\; & \; \end{array}$$

Note that $\frac{k\sigma_{T}^{2}+\sigma_{e}^{2}}{\sigma_{e}^{2}}=k\left(\frac{\sigma_{T}^{2}}{\sigma_{e}^{2}}\right)+1=\text{kF}+1$ The variance is given as

$$\begin{array}{lll} \text{Var}(\hat{F}) & =\left[\frac{n(k-1)-2}{k^{2}(n-1)}\right]\; & \;\\ & \;\times \left[\frac{n+1}{n(k-1)-4}-\frac{n-1}{n(k-1)-2}\right]{\left(\text{kF}+1\right)}^{2}\; & \; \end{array}$$

## Competing interests

The authors declare that they have no competing interests.

## Authors’ contributions

EGA contributed to the conception and design of the study, analysis and interpretation of data and drafting of the manuscript. JSH contributed to the conception and design of the study, interpretation of data and drafting of the manuscript. TT, ARW and BMF helped with critical revision of the manuscript for important intellectual content. JB contributed to the conception and design of the study, interpretation of data and drafting of the manuscript. All authors read and approved the final manuscript.

## Pre-publication history

The pre-publication history for this paper can be accessed here:

http://www.biomedcentral.com/1471-2288/12/126/prepub
